# Probing Subcellular Iron Availability with Genetically Encoded Nitric Oxide Biosensors

**DOI:** 10.3390/bios12100903

**Published:** 2022-10-21

**Authors:** Gulsah Sevimli, Amy E. Alston, Felix Funk, Beat Flühmann, Roland Malli, Wolfgang F. Graier, Emrah Eroglu

**Affiliations:** 1 Molecular Biology, Genetics and Bioengineering Program, Faculty of Engineering and Natural Sciences, Sabanci University, Istanbul 34956, Turkey; 2 Department of Molecular Biology and Biochemistry, Gottfried Schatz Research Center, Medical University of Graz, 8010 Graz, Austria; 3 CSLVifor Ltd., Redwood City, CA 94063, USA; 4 CSL Vifor Ltd., Flughofstrasse 61, CH-8152 Glattbrugg, Switzerland; 5 BioTechMed Graz, Mozartgasse 12/II, 8010 Graz, Austria; 6 Next Generation Fluorescence Imaging Inc., 8010 Graz, Austria; 7 Research Institute for Health Sciences and Technologies (SABITA), Istanbul Medipol University, Istanbul 34810, Turkey

**Keywords:** labile iron, cellular iron uptake, fluorescent biosensor, geNOps, Ferinject, Venofer, carboxymaltose, iron sucrose, HBED

## Abstract

Cellular iron supply is required for various biochemical processes. Measuring bioavailable iron in cells aids in obtaining a better understanding of its biochemical activities but is technically challenging. Existing techniques have several constraints that make precise localization difficult, and the lack of a functional readout makes it unclear whether the tested labile iron is available for metalloproteins. Here, we use geNOps; a ferrous iron-dependent genetically encoded fluorescent nitric oxide (NO) biosensor, to measure available iron in cellular locales. We exploited the nitrosylation-dependent fluorescence quenching of geNOps as a direct readout for cellular iron absorption, distribution, and availability. Our findings show that, in addition to ferrous iron salts, the complex of iron (III) with N,N’-bis (2-hydroxybenzyl)ethylenediamine-N,N’-diacetic acid (HBED) can activate the iron (II)-dependent NO probe within intact cells. Cell treatment for only 20 min with iron sucrose was also sufficient to activate the biosensor in the cytosol and mitochondria significantly; however, ferric carboxymaltose failed to functionalize the probe, even after 2 h of cell treatment. Our findings show that the geNOps approach detects available iron (II) in cultured cells and can be applied to assay functional iron (II) at the (sub)cellular level.

## 1. Introduction

Iron is an essential transition metal for the human body, most of which can be found in the blood circulatory system and muscles [1]. In cells, only a fraction of iron is complexed in metalloproteins or soluble iron (labile iron) [2]. Major cellular iron uptake pathways are: (i) via transferrin receptors TfR1 and TfR2, which allow for the entry of ferric iron into lysosomes, where it can be reduced to ferrous iron to be delivered to the cytosol through divalent cation transporter (DMT1). (ii) DMT1 channels are also on the cell surface, where iron can enter the cell in its reduced form through the cell plasma membrane into the cytosol, available as labile iron [3]. Cleverly designed nanoparticles of polynucleare iron carbohydrate complex were developed for intravenous iron therapy [4,5]. In contrast, upon therapeutic administration, these intravenous iron–carbohydrate complexes are opsonized by macrophages and delivered to the principal organs of the mononuclear phagocytic system, the liver, and the spleen [4]. Iron is then delivered to the bone marrow via normal iron transport systems [5]. Cells have several biochemical strategies to control the labile ferrous iron pool [6]. Ferritin, a protein complex found in every cell type, can oxidize and store vast amounts of iron [7]. The reduction and release of iron from ferritin in a controllable physiological manner seems to depend on the cellular lysosomal activity but might also involve other iron-reducing and mobilization mechanisms [8]. Some cells express ferroportin−1, a transmembrane protein, which can efficiently export iron from cells [2]. However, labile iron in excess has adverse effects. It can trigger the generation of oxygen radicals and reactive molecules, including hydroxyl radicals (OH^−^), superoxide anions (O_2_^−^), and hydrogen peroxide (H_2_O_2_) [9]. These reactive oxygen species (ROS) may cause many diseases, such as cancer and neurodegenerative and cardiovascular diseases [9,10]. Thus, there is a particular interest in studying the labile iron pool in cells to understand better the causes and consequences of ferrotoxicity [11]. 

Iron bioimaging is challenging, and the soluble iron pool is poorly characterized due to the lack of suitable tools. Conventional methods such as magnetic resonance imaging (MRI) lack spatial resolution, while other techniques are incapable of live-cell imaging (Perls’ Prussian Blue Staining with 3,3’-diaminobenzidine (DAB) enhancement) [12,13]. Modern approaches employ fluorescent probes; fluorescein-based probes are Calcein [14] and PhenGreen-SK [15], which are fluorescence quenching-based probes with greater sensitivity for ferrous iron over ferric iron. However, these probes display poor selectivity and are responsive to other metals, undermining their selective applicability. RhoNox−1 probes are the first fluorescence turn-on probes, with more excellent selectivity for ferrous iron [16]. However, significant drawbacks are the localization of the probe to the Golgi apparatus and disabling precise localization of labile iron. Newer multicolored versions with improved characteristics and differentially targeted chemical-based probes have been devised (Mem-RhoNox, CoNox-1, FluNox-1, and SiRhoNox-1) [17]. A recent study developed a bioluminescent-based probe termed iron-caged luciferin-1 (ICL-1) that has been used to in vivo image labile iron in a physiologically relevant concentration range [18]. ICL-1 revealed, in a mouse model of systemic bacterial infection, increased iron increase in infected tissues. However, the lack of sensitivity, ultra-local targetability, pH dependency, and the lack of biochemical readouts of these probes undermine the precise evaluation of bioavailable cellular iron. 

The geNOps, genetically encoded nitric oxide (NO) biosensors, are non-heme iron-containing metalloproteins, and their functionality requires iron (II) [19]. Ferrous but not ferric iron can activate the probe [20,21,22,23,24]. In a recent study, we exploited the geNOps technology to study the role of pericellular oxygen levels and cellular ferrous iron uptake [25]. The study unveiled that cells cultured under physiological levels of oxygen (5 kPa O_2_) require lower iron (II) supplementation compared to cells adapted to hyperoxia (18 kPa O_2_). We concluded from these results that the geNOps biosensor indirectly reports intracellular iron levels yet did not further tackle this feature of the probes. geNOps are genetically encoded nitric oxide (NO) biosensors and metalloproteins consisting of a bacteria-derived non-heme iron-binding GAF domain [26]. The ferrous iron within the GAF domain can be nitrosylated, resulting in a measurable fluorescence quench with high sensitivity, selectivity, and reversibility [19]. In this study, we tested the iron (II) dependency of geNOps and exploited this critical feature as an intracellular iron biosensor using cultured cells stably expressing the orange variant of geNOps. To demonstrate the suitability of our approach, we assayed the efficacy of three different groups of iron compounds, including iron salts, a ferric iron chelate complex, and polynuclear iron (III)–hydroxide carbohydrate complexes [27,28]. Our study provided a potentially novel iron-compound screening assay in cultured cells to study iron uptake and its bioavailability.

## 2. Materials and Methods

### 2.1. Cell Culture

HEK293T and HeLa cells were maintained in Dulbecco’s modified eagle medium (DMEM) supplemented with 10% fetal bovine serum (FBS) (Pan-Biotech, Aidenbach, Germany), 100 µg/mL Penicillin (Pan-Biotech, Aidenbach, Germany), and 100 U/mL Streptomycin (Pan-Biotech, Aidenbach, Germany). EA.hy926 cells (ATCC, CRL-2922, University Blvd, VA, USA) were cultured under the same conditions as HEK293T and HeLa cells but additionally supplemented with 100 µg/mL Normocin (InvivoGen, San Diego, CA, USA) and 2% HAT ((Sodium Hypoxanthine (5 mM), Aminopterin (20 µM), and Thymidine (0.8 mM)) (ATCC, USA). Cells were maintained in a humidified CO_2_ incubator (5% CO_2_, 37 °C). Stable EA.hy926 ] and HEK293T cell lines expressing O-geNOps-NES [24] used in this study were developed in previous studies [25]. The same protocols were applied to generate stable HeLa cells, as described previously [29]. Cells with Mito-C-geNOps, stably O-geNOps-NES expressing HeLa, were seeded on 30 mm glass coverslips for transient transfection. 16–24 h later, 1 µg of each purified plasmid was transfected using a 2.5 µL PolyJet transfection reagent according to the manufacturer’s instructions.

### 2.2. Experimental Buffers

If not otherwise stated, all chemicals were purchased from NeoFroxx. To maintain cells outside of the incubation chamber, a cell storage buffer containing 2 mM CaCl_2_, 5 mM KCl, 138 mM NaCl, 1 mM MgCl_2_, 1 mM 4-(2-hydroxyethyl)-1-piperazineethanesulfonic acid (HEPES) (Pan-Biotech, Aidenbach, Germany), 0.44 mM KH_2_PO_4_, 2.6 mM NaHCO_3_, 0.34 mM NaH_2_PO_4_, 10 mM D-Glucose, 0.1% minimum essential medium (MEM) Vitamins (Pan-Biotech, Aidenbach, Germany), 0.2% essential amino acids (Pan-Biotech, Aidenbach, Germany), 100 µg/mL Penicillin (Pan-Biotech, Aidenbach, Germany) and 100 U/mL Streptomycin (Pan-Biotech, Aidenbach, Germany) was used. The pH was adjusted to 7.43 using 1 M NaOH. The cell storage buffer was sterilized using a 0.45 µm medium filter (Isolab, Eschau, Germany). For live-cell imaging experiments, a HEPES-buffered solution was used, consisting of 2 mM CaCl_2_, 5 mM KCl, 138 mM NaCl, 1 mM MgCl_2_, 10 mM HEPES, 10 mM D-Glucose, and pH was adjusted to 7.43 using 1 M NaOH.

### 2.3. Cellular Iron Loading Procedure 

**Figure 1:**
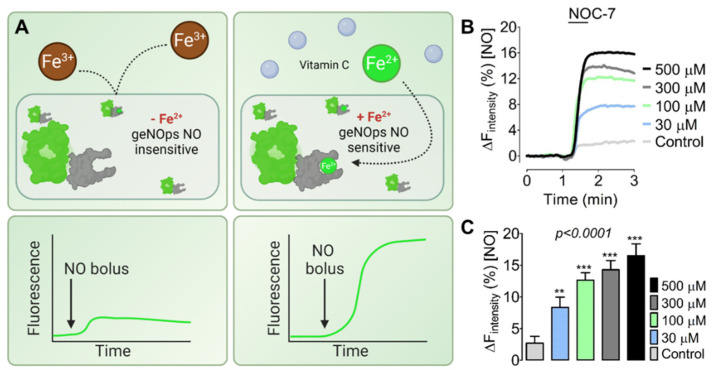
NO biosensor reports labile iron (II) in a concentration-dependent manner. (**A**) The cartoon shows iron (II) dependency of the NO biosensor geNOps. If iron salts are provided to cells, only ferrous iron can enter the cell cytosol through DMT1 channels but not ferric iron. Non-heme iron consisting of geNOps can sense NO in an iron (II)-concentration-dependent manner. (**B**) Average curves show real-time NO signals in response to 10 μM NOC-7 in HEK293T cells expressing O-geNOps. Cells have been treated with equimolar concentrations of FeSO_4_ and Vitamin C, respectively, for 20 min before imaging experiments: 500 μM (black curve, *n* = 3/60), 300 μM (dark grey curve, *n* = 3/54), 100 μM (green curve, *n* = 3/56), 30 μM (blue curve, *n* = 3/42), and control no treatment (light grey curve, *n* = 3/73). (**C**) Bars represent the statistical analysis of iron (II) dependent geNOps responses shown in panel (**B**). All values are given as ±SD, and Dunnet’s multiple comparison test was applied. ** 0 vs. 30 um, Mean Difference (Diff): −5.525, q = 5.182, 95% Confidential Interval (CI) of Diff: −8.607 to −2.443; *** 0 vs. 100 um; Mean Diff: −9.870; q = 9.257, 95% CI of Diff: −12.95 to −6.79; *** 0 vs. 300 um; Mean Diff: −11.400; q = 10.69, 95% CI of Diff: −14.48 to −8.32; *** 0 vs. 500 um; Mean Diff: −13.800; q = 12.94, 95% CI of Diff: −16.88 to −10.71.

**Figure 2:**
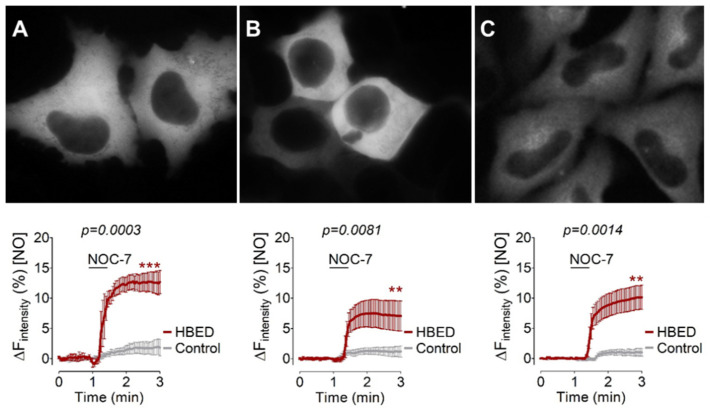
Evaluation of the ferric iron chelator HBED and its effectiveness in different cultured cells to activate geNOps. (**A**) Representative wide-field images show immortalized endothelial cells (Ea.hy926), (**B**) human embryonic kidney cells (HEK293T), and (**C**) cervical cancer cells (HeLa) expressing cytosolic O-geNOps. *Lower Panels:* Average curves show NO responses of cells in response to 10 μM NOC-7 that have been treated with equimolar concentrations of HBED and Vitamin C, 1 mM, respectively, for 20 min before imaging experiments. The left panel shows Ea.hy926 cells (control grey curve, *n* = 3/60; HBED-treated, red curve, *n* = 3/74), middle panel HEK293T cells (control grey curve, *n* = 3/54; HBED-treated, red curve, *n* = 3/66) and the right panel shows HeLa cells (control grey curve, *n* = 3/29; HBED-treated, red curve, *n* = 3/18). All values are given as mean ± SD, and Student’s *t*-test was applied to compare maximum amplitudes between HBED-treated cells and control cells. (**A**) *** *p* = 0.0003, (**B**) ** *p* = 0.0081, (**C**) ** *p* = 0.0014.

**Figure 3:**
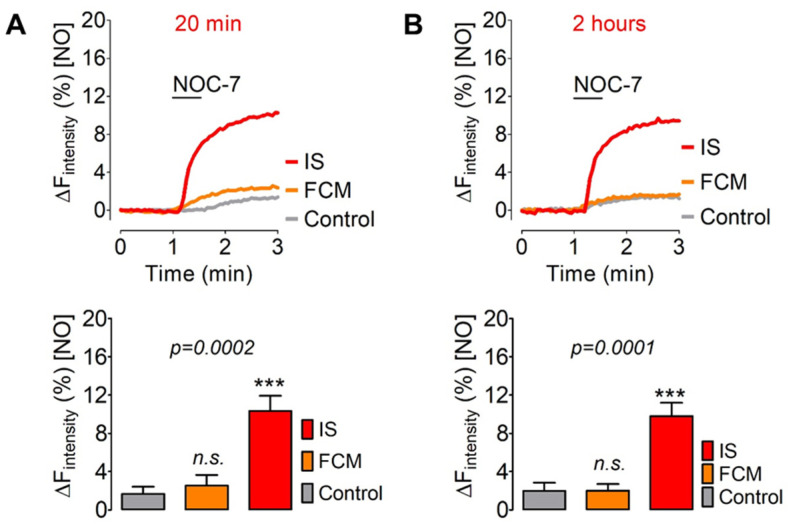
Evaluation of the effectiveness of IS and FCM in cultured Eahy.926 cells to activate geNOps. (**A**) The upper panel shows representative real-time traces of intracellular NO signals in response to 10 μM NOC-7 reflecting the labile iron pool in Ea.hy926 cells upon treatment with IS (red curve) or FCM (orange curve) vs. control cells (grey curves). Cells were pretreated with 500 μg iron/mL with the respective drug for 20 min before the imaging experiment. The lower panel shows statistical analysis. Control (grey curve, *n* = 3/46), FCM (orange curve, *n* = 3/47), and IS (red curve, *n* = 3/55). Panel (**B**) shows the same experimental setup as in panel (**A**), with the difference that cell pretreatment with IS and FCM was extended to 2 h. Control (grey curve, *n* = 3/29), FCM (orange curve, *n* = 3/28), and IS (red curve, *n* = 3/32). All values are given as mean ± SD, and Dunnet’s multiple comparison test was applied. (**A**) *** *p* = 0.0002, (**B**) *** *p* = 0.0001.

**Figure 4:**
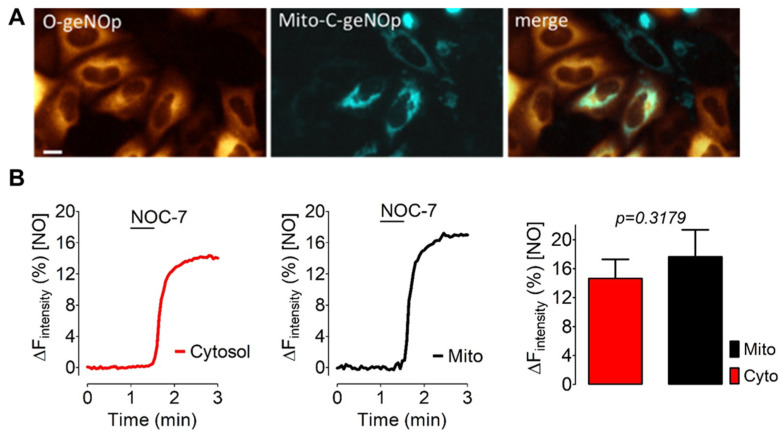
Assessing iron (II) levels in subcellular locales upon IS treatment. (**A**) Representative wide-field images show HeLa cells stably expressing O-geNOps-NES (orange) transiently transfected with mitochondria-targeted C-geNOps (Mito-C-geNOp, cyan). (**B**) Real-time traces of intracellular NO signals in response to 10 μM NOC-7 in the cell cytosol (red curve and bar, co-imaged with O-geNOps, *n* = 3/18) or mitochondria (black curve and bar, co-imaged with C-geNOps, *n* = 3/18). All values are given as mean ± SD, and Student’s *t*-test was applied to compare maximum amplitudes between mitochondria and cytosolic NO signals.

Equimolar concentrations of FeSO_4_ or the iron complex with the hexadentate phenolic amino carboxylate iron chelator HBED and Vitamin C in a HEPES-buffered physiological salt solution (see Section 2.2. for imaging media) were used to pretreat cells for 20 min at 5% CO_2_ and 37 °C. Cells were washed twice with PBS to remove excessive iron (II) from cells before the imaging experiment (this protocol was used for Figure 1 and Figure 2). 500 μg iron/mL iron sucrose (Venofer^®^, IS, Vifor Pharma, Glattbrugg, Switzerland) or ferric carboxymaltose (Ferinject^®^, FCM, Vifor Pharma, Glattbrugg, Switzerland) were diluted in DMEM to incubate cells for 20 min or 2 h at 5% CO_2_ and 37 °C. Cells were washed twice with PBS to remove excessive iron (II) from cells before the imaging experiment (this protocol was used in Figure 3 and Figure 4). 

### 2.4. Imaging Experiments

Live-cell imaging experiments were performed on an inverted wide-field epi-fluorescence microscope Zeiss Axio Observer.Z1/7 (Carl Zeiss AG, Oberkochen, Germany) equipped with an LED light source Colibri 7 (423/44 nm, 469/38 nm, 555/30), Plan-Apochromat 20x/0.8 dry objective, Plan-Apochromat 40x/1.4 oil immersion objective, a monochrome C.C.D. camera Axiocam 503, and a custom-made gravity-based perfusion system. O-geNOps signals were imaged by exciting cells (555 nm) using a motorized dual filter wheel equipped with the filter combinations FT570 (BS) and emission filter 605/70. Control and data acquisition were executed using Zen Blue 3.1 Pro software (Carl Zeiss AG, Oberkochen, Germany). Administration and withdrawal of 3-(2-Hydroxy-1-methyl-2-nitrosohydrazino)-N-methyl-1-propanamine (NOC-7) were performed using a custom-made perfusion system connected to a metal perfusion chamber (NGFI, Graz, Austria).

### 2.5. Statistics

Image analysis was conducted using GraphPad Prism software version 5.04 (GraphPad Software, San Diego, CA, USA). Throughout, all experiments were repeated at least in triplicate. Experimental repeats are given ‘N’, whereby the number of cells imaged is indicated as ‘*n*’. For instance: 3/21 indicates N = 3 (triplicate) and *n* = 21 (number of cells imaged on a single culture dish). All statistical data are presented as mean ± SD in addition to the representative real-time traces shown as curves (if not indicated otherwise). For the statistical comparisons of multiple groups, one-way ANOVA analyses of variances with post-test Dunnet’s multiple comparison test (comparison of all columns against control column; data in Figure 1C and Figure 3A,B lower panels) or Student’s *t*-test (unpaired; data in Figure 2A–C lower panels and Figure 4B right panel) were used to compare two experimental conditions with each other. Statistical significances were considered significant and indicated with “*” and respective *p*-values are given in the figure. geNOps signals are based on fluorescence quenching, and for a more straightforward representation, geNOps responses have been inverted as described elsewhere [19,20] using the formula ΔF_Intensity_ = (1 − (F/F_0_) × 100). 

## 3. Results

### 3.1. Characterizing geNOps’ Ferrous Iron Dependency

From former studies [30,31,32], we concluded that genetically encoded fluorescent NO biosensors, referred to as geNOps, in cultured cells require extracellularly supplied iron (II) to become fully active, i.e., NO-sensitive (Figure 1A). To correlate geNOps’ functionality with increasing levels of iron (II), we performed NO imaging experiments with stable cytosolic O-geNOps expressing HEK293T cells that we pretreated with different concentrations of equimolar iron (II) sulfate and vitamin C for 20 min. We then washed and kept cells in an iron-free storage buffer before live-cell imaging. Figure 1B shows that administering the same levels of exogenous NOC-7 evoked more robust O-geNOp signals when cells were treated with higher concentrations of iron (II) sulfate and vitamin C. This approach documented that the intracellular biosensor’s functionality strongly depends on the provided extracellular iron (II) concentration (Figure 1C). This critical feature of geNOps permits the assessment of bioavailable intracellular ferrous iron levels and may be exploitable to assay cellular iron uptake in live cells.

### 3.2. Imaging Chelated Ferric Iron Bioavailability

Next, we challenged our hypothesis of whether geNOps might be useful as an intracellular reporter for available iron from a ferric iron complex. For this purpose, we tested three different cultured cell lines stably expressing O-geNOps (Ea.hy926, HEK293T, and HeLa) (Figure 2). We incubated these cells for 20 min with iron (III)-HBED complex. Ferric iron forms a very stable complex with HBED [33], which might release ferrous iron in the presence of vitamin C. In former studies, we have shown that geNOps cannot incorporate ferric iron—if ferric iron salts are provided—and remain silent in response to NO administration [19]. However, all three cell lines pretreated with iron (III)-HBED and vitamin C yielded a robust O-geNOp signal when exposed to extracellular NO (Figure 2). Interestingly, the effect was most pronounced in the endothelial cell line (Figure 2A) compared to HEK293T (Figure 2B) and HeLa (Figure 2C) cells.

### 3.3. Comparing the Intracellular geNOps Activation with FCM and IS

We next tested our approach with iron carbohydrate nanoparticles. For this purpose, we performed a comparative test of ferric carboxymaltose (FCM) and iron sucrose (IS) in cultured endothelial cells. Both short (20 min) (Figure 3A) and long-term (2 h) (Figure 3B) cell treatment with FCM and IS yielded similar results. IS robustly activated geNOps functionality, indicating a fast and sustained elevation of available ferrous iron in endothelial cells in response to the sucrose-based iron complex. FCM, under the same experimental conditions, failed to activate the biosensor’s NO responsiveness (Figure 3).

### 3.4. IS Supplies Ferrous Iron Also to Mitochondria of Cancer Cells

To test whether IS also supplies ferrous iron to mitochondria we expressed the mitochondria-targeted cyan variant of geNOp, termed Mito-C-geNOp, in HeLa cells stably expressing cytosolic O-geNOps. Most cells were positive for both geNOps variants (Figure 4A). The distinct spectral properties of both biosensors allowed for us to simultaneously monitor geNOps signals in both compartments, the cell cytosol in the orange channel and mitochondria in the cyan channel. While untreated control cells showed only marginal geNOps signals in both compartments in response to NOC-7, the geNOps signals in cells pretreated with IS for 2 h rapidly plateaued in the cytosol and mitochondria (Figure 4B). These data indicate that geNOps also permit the detection of ferrous iron in subcellular locales such as mitochondria. Moreover, these data show that cell treatment with IS supplies the cytosol and mitochondria with bioavailable iron.

## 4. Discussion

The present study investigating iron-dependent biosensor functionality in cultured cells demonstrates that the geNOps reliably assay ferrous iron availability in subcellular locales. Our experiments unveiled that, upon adding a potent NO-donor, geNOps response help with the direct measurements of the intracellular ferrous iron bioavailability. Exploiting this feature of the NO biosensor, we observed that cultured cells could maintain their intracellular iron (II) pool with different iron compounds, including a ferrous iron salt, a ferric iron chelate complex, and ferric iron–hydroxide carbohydrate complexes. This method might be informative for further studies that aim to test the iron-loading efficiency of diverse iron formulations and experimental conditions to correlate the biochemical consequences of short- and long-term iron treatment.

The ferrous iron dependency of geNOps is essential because iron (II) bound to the non-heme iron center of the bacterial GAF-domain in the biosensor allows for its nitrosylation, which turns into a measurable fluorescence signal in response to NO [19]. Notably, this event depends on the amount of iron provided to the cells exogenously (Figure 1). However, it has not been feasible to control exact concentrations of iron (II) to calibrate geNOps in cells. Additionally, the calibration of recombinant geNOps failed [25]. Thus, the assay only assesses subcellular iron availability, but does not provide an absolute quantification of subcellular iron (II) concentrations.

We show that the assay requires the addition of an NO donor to unmask the iron (II)-dependency of the NO-induced fluorescent quench. NO is highly lipid-soluble and can easily cross biomembranes [34]. Thus, he exogenous administration of a NO-liberating compound immediately nitrosylates metalloproteins in subcellular locales, including the mitochondria-located geNOps. Exploiting the geNOps response as a readout for subcellular iron levels, we showed that cultured cells’ iron (II) bioavailability in vitro was too low to functionalize the biosensor efficiently (Figure 1). In all tested cell lines (HEK293, HeLa, and EA.hy926 cells), the NOC-7-induced geNOps signal was negligible (Figure 2). This observation indicates that the overexpressed NO biosensor proteins are insufficiently supplied with iron (II) when cells are kept in standard cell culture media without additional iron administration. Commercially available cell culture media (i.e., DMEM) contain little (0.25 µM) ferric iron salts. Cells cultured under standard cell culture conditions are often supplied with fetal calf serum (FCS), which might contain unknown levels of iron (III)-loaded transferrin and ferritin [35].

Nevertheless, a standard cell culture medium with 10% FCS was suggested to contain no more than 5 µM iron [36]. Thus, it seems questionable if cells cultured under such conditions might have adapted to a low iron supply to counteract and survive a cellular iron deficiency. However, cell treatments with iron-chelating compounds such as deferoxamine, which has the potency to further reduce the cellular iron content, have detrimental effects, pointing to the importance of a minimal cellular iron supply for cellular growth and viability [36]. The iron needed to drive cell proliferation and maintain cell viability in vitro seems very low [37].

It is worth noting that fluorescence imaging of geNOps was performed in the absence of iron compounds. Cells were iron-treated before the imaging experiments; thus, the experimenter only retrieved the information on the cellular iron status once the NO-donor was administered to cells expressing the NO biosensor. Thus, it stands to reason that geNOps have an “iron memory”, which depends on the availability of the ferrous iron bond to the GAF domain in geNOps. Further experiments are necessary to better understand this memory and the factors that control it by oxidizing or removing the iron (II) ion from the non-heme iron center of the NO biosensor. We could not determine if the geNOps assay reports subcellular iron alterations reversibly. The NO-dependency of the assay can also complicate its correct usage and data interpretations if the NO molecule is produced endogenously. In cells with high rates of basal endogenous NO biosynthesis, such as macrophages, pharmacological tools such as nitric oxide synthase (NOS) inhibitors and NO scavengers might be helpful. Under such conditions, a fluorescent iron biosensor, which directly and NO-independently responds to iron binding with a fluorescence signal seems advantageous. The geNOps design might facilitate such future developments.

Nevertheless, this study confirms that the soluble ferrous iron provision to cells, which likely enters the cells via plasma membrane located divalent metal transporter 1 (DMT1), results in the functionalization of geNOps in a concentration-dependent manner (Figure 1). We also showed, for the first time, that complexed iron (III) formulations, including iron (III)-HBED and ferric iron–hydroxide carbohydrate complexes, also activated geNOps. We do not yet understand if these findings indicate the cellular uptake, biodegradation, and reduction of the iron (III) complexes. We cannot rule out the possibility that minor amounts of ferrous iron in the complexes or extracellular reduction of iron (III) to iron (II) accounts for intracellular functionalization of the NO biosensor by ferrous iron supply under these conditions. Additional experiments are required to understand by which molecular mechanisms the different iron (III) formulations contribute to cellular iron (II) bioavailability. In follow-up studies, we plan to investigate the biochemical and functional consequences of cellular iron elevations that can be achieved with the iron (III) complexes.

Our studies also unveiled that ferric iron nanoparticles have different potencies to supply ferrous iron to geNOps. In contrast to FCM, cell treatment with IS fully activated the geNOps biosensor (Figure 3). These results are consistent with recent studies that suggest that IS and FCM [4] have different pharmacokinetic profiles, possibly owing to variations in the rate and amount of iron absorption and biodegradation to bioavailable iron. The current research aims to understand better the impact of the carbohydrate ligand of such iron (III)-containing complexes on their biological activity [5]. Different carbohydrates, including disaccharides, oligosaccharides, and polysaccharides, have been tested as an alternative pharmacological approach to improve iron nanoparticles’ safety and efficacy [4,5]. Considering that the geNOps technology even allows the detection of the distribution of intracellular iron levels (Figure 4), this approach may potentially open new avenues in analytical nanomedicine to investigate subcellular drug-targeting and for application to bioequivalence evaluation strategies [5,37].

In conclusion, our findings show that the geNOps technology offers great potential to understand iron-containing drugs’ different pharmacological and pharmacodynamic properties in vitro. Future studies in our laboratories aiming to establish intravital geNOps imaging may help to elucidate the role of iron metabolism in vivo.

## Data Availability

Data are available upon reasonable request.
